# Unveiling the Intrusion: An Emergent Journey Into Tuberculous Pericarditis

**DOI:** 10.7759/cureus.65093

**Published:** 2024-07-22

**Authors:** Ashmin Singh, Enrique C Bernal, Shakeera Dunn, Ramesh Vemulapalli

**Affiliations:** 1 Internal Medicine, Bayhealth Hospital, Dover, USA; 2 Infectious Disease, Bayhealth Hospital, Dover, USA

**Keywords:** ripe, acid-fast bacillus, pericardial diseases, pericarditis, tuberculosis

## Abstract

We present an interesting case of mycobacterial tuberculosis pericarditis presenting as effusive constrictive pericarditis with early cardiac tamponade in a young Mexican migrant of Haitian descent. The patient underwent a pericardial window and was treated with rifampin, isoniazid, pyrazinamide, ethambutol, and vitamin B6. After further receiving steroids, the patient was doing well and was discharged home safely.

## Introduction

Tuberculosis, caused by *Mycobacterium tuberculosis*, continues to be a healthcare crisis in many parts of the world. Even though it primarily causes a pulmonary pathology, it can disseminate in different organs of the body, leading to different manifestations of the infection. One of the manifestations of tuberculosis is tuberculosis pericarditis, in which the infection affects the pericardium, the sac that surrounds the heart. Symptoms associated with pericarditis are vague and nonspecific such as dyspnea, fever, and cough [1]. However, in rare circumstances, tuberculosis pericarditis can worsen into cardiac tamponade, which results in rapid accumulation of pericardial fluid leading to hemodynamic instability. Only 1-2% of patients with pulmonary tuberculosis develop tuberculosis pericarditis [2]. Here, we present an interesting case of mycobacterial tuberculosis (M. tb) pericarditis presenting as effusive constrictive pericarditis with early cardiac tamponade in a young Mexican migrant of Haitian descent. This case highlights the importance of imaging needed to ensure early recognition and timely intervention.

## Case presentation

**Figure 1:**
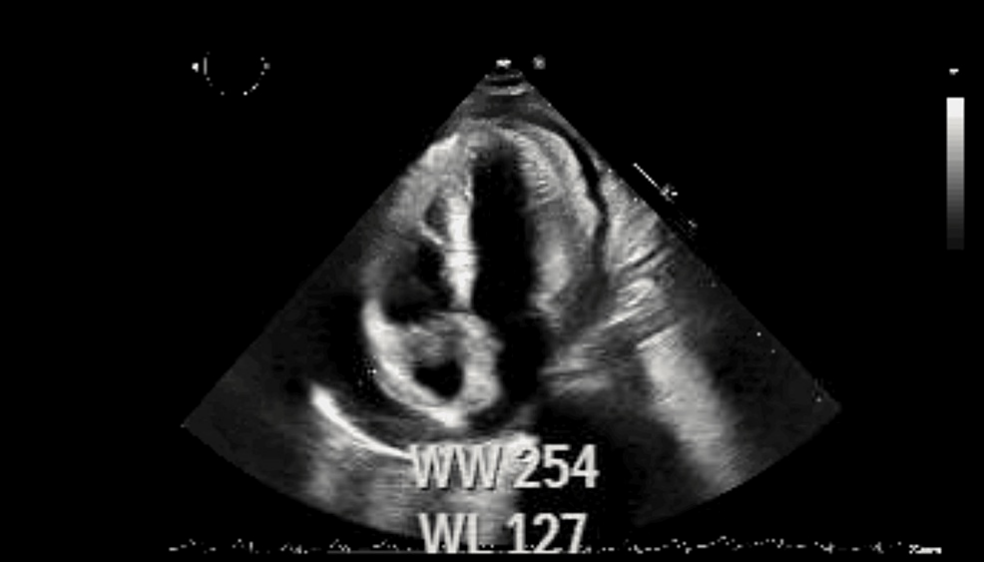
The early signs of cardiac tamponade of right-sided heart collapse are shown in the echocardiogram.

A 30-year-old human immunodeficiency virus (HIV)-negative man, a recent immigrant from Mexico of Haitian descent with no significant past medical history, presented to the emergency department with complaints of shortness of breath and palpitations for two weeks. On presentation, he was found to be tachycardic with a heart rate of around 150. A two-dimensional (2D) echocardiogram showed small pericardial effusion (<1 cm) and abnormal septal motion, suggestive of pericardial constriction (Figure 1).

**Figure 2:**
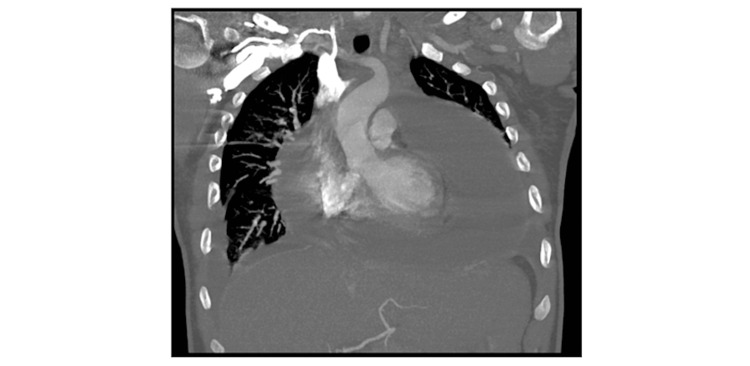
The CT chest shows a large pericardial effusion around the heart.

A chest CT scan revealed an extensive complex pericardial effusion (Figure 2).

He underwent a pericardial window via a subxiphoid approach in which the incision was made and a needle inserted into the retroxiphoid space and advanced into the pericardium. A wire was later inserted and the 14-Fr pigtail catheter was positioned over the wire and advanced to remove excess fluid. About 1,500 mL of fluid was removed around the pericardium during the procedure. Six days later, the patient became tachycardic again with high-grade fevers, and a repeat 2D echocardiogram also began to show signs of early cardiac tamponade. The patient again underwent a pericardial window with the subxiphoid approach and was found to have a very thickened pericardium this time, making sarcoidosis and tuberculosis high on the differential. The patient had about 200 to 300 mL of fluid removed with a Blake drain placed.

**Figure 3:**
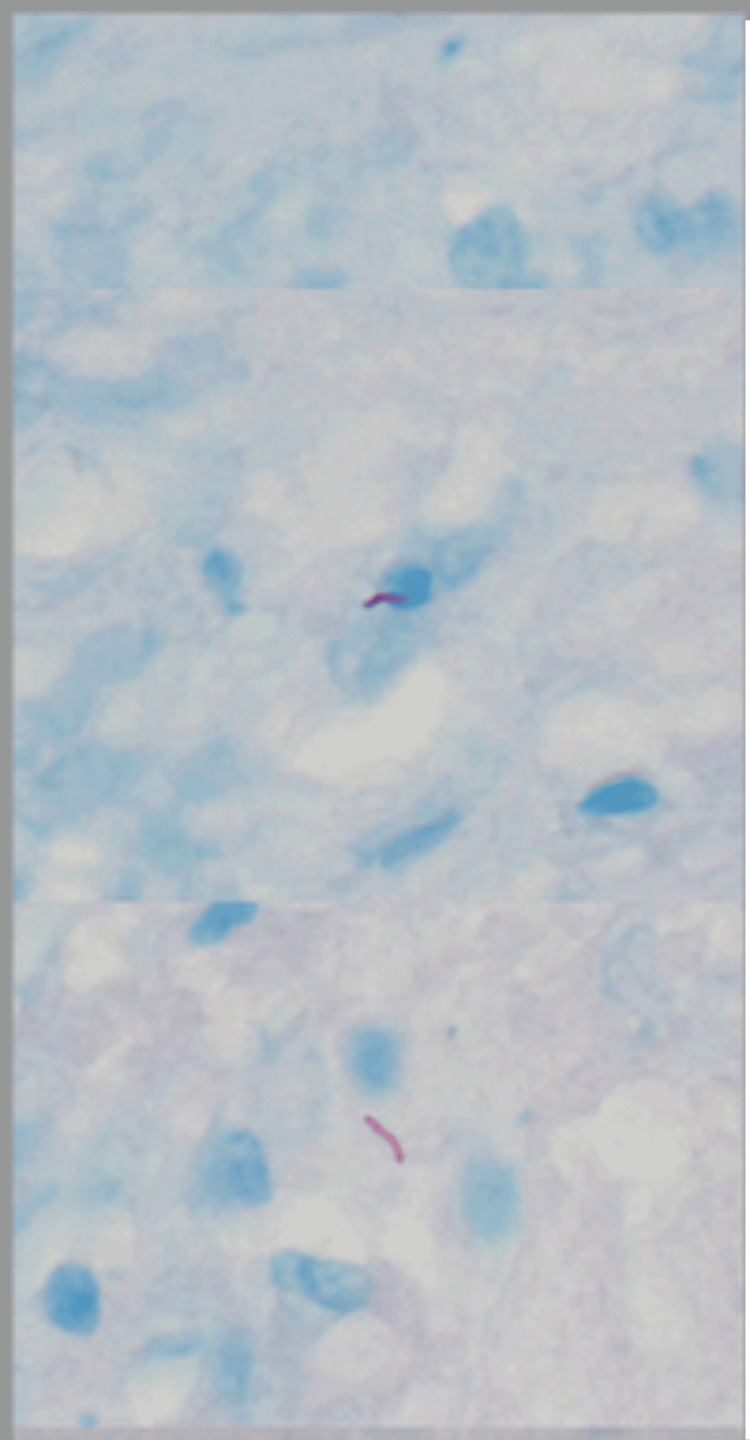
The pericardial tissue biopsy showing multiple acid-fast bacilli-positive organisms.

The cultures and cytology were not back by the time of the two procedures. However, the pericardial fluid cultures grew acid-fast bacilli (AFB) in broth culture, and M. tb was detected by polymerase chain reaction (PCR). Pleural fluid adenosine deaminase was elevated, but the AFB smear and cultures were negative. Pericardial biopsy and histopathology revealed fibrinous pericarditis with occasional poorly formed granulomata, and AFB stain demonstrated several AFB, histologically consistent with *Mycobacterium* species. Pericardial tissue cultures grew AFB in broth (Figure 3), and M. tb was detected by PCR.

After the pericardial tissue biopsy revealed AFB, the patient was started on rifampin, isoniazid, pyrazinamide, ethambutol (RIPE), and vitamin B6 for nine months. He was also given adjunctive steroids. The patient was started on prednisone 40 mg PO daily for four weeks, 30 mg for two weeks, and 15 mg PO daily for one week, followed by 5 mg for the last week. The patient received prednisone for a total of 56 days. He was discharged home in stable condition with close follow-up by the local county tuberculosis clinic.

## Discussion

Pericarditis caused by M. tb is seen in 1% of all autopsied cases of tuberculosis and 1-2% of cases with pulmonary tuberculosis [3]. However, the incidence is higher in countries that are highly endemic for tuberculosis, such as Sub-Saharan African countries, with a high prevalence of HIV. Prompt diagnosis and treatment are important as the median survival of untreated tuberculosis pericarditis is 3.7 months [3]. Early diagnosis is difficult, with a mean interval of 5.2 weeks between hospital admission to establishing the diagnosis (range: 1-14 weeks)[3]. Here, we present the case of tuberculosis pericarditis and pericardial effusion with a diagnosis established within seven days of hospital admission. Our case is considered a definitive diagnosis of tuberculosis pericarditis based on the demonstration of AFB consistent with M. tb in pericardial fluid and pericardial tissue samples. Once granulomas and AFB were seen on the histopathology of pericardial tissue, we initiated anti-tuberculosis treatment with RIPE. We used adjunctive corticosteroids in our patient based on limited data that suggest steroids reduce overall mortality and the development of constrictive pericarditis. However, these studies had several limitations, including a small sample size and being done in the pre-HIV era. Therefore, routine use of corticosteroids to treat tuberculosis pericarditis cannot be recommended, and further studies are needed to assess their effectiveness in treating tuberculosis pericarditis.

## Conclusions

Tuberculosis pericarditis continues to remain a significant challenge in endemic regions and timely recognition is required for effective treatment and management. Further research is required for improved outcomes in patients with pericarditis.
